# Aripiprazole lai two-injection start in a 16 year-old with Schizophrenia

**DOI:** 10.1192/j.eurpsy.2022.2058

**Published:** 2022-09-01

**Authors:** B. Marpepa, C. Appignanesi, L. Orsolini, U. Volpe, V. Salvi

**Affiliations:** 1 Politechnic University of Marche, Department Of Neuroscience/dimsc, Ancona, Italy; 2 Polytechnic University of Marche, Department Of Clinical Neurosciences/dimsc, School Of Medicine, Unit Of Psychiatry, Ancona, Italy; 3 Università Politecnica delle Marche, Unit Of Clinical Psychiatry, Department Of Clinical Neurosciences/dimsc, Ancona, Italy

**Keywords:** schizophrénia, lai two-injection, Psychofarmacology, Psychosis

## Abstract

**Introduction:**

Aripiprazole LAI is approved for the treatment of schizophrenia in adults. Recently, Europe and Canada approved the use of the two-injection start (TIS) regimen: two separate injections of 400 mg long-acting aripiprazole along with a single 20 mg dose of oral aripiprazole. Aripiprazole showed efficacy in the treatment of adolescents with acute schizophrenia in several controlled trials, leading to its approval for 13-17 year-old adolescents with schizophrenia by the EMA. However, the LAI formulation still remains off-label in adolescents.

**Objectives:**

To demonstrate the efficacy and safety of the TIS regimen of aripiprazole LAI formulation in a 16 year-old adolescent with schizophrenia.

**Methods:**

We evaluated the symptoms of schizophrenia and general severity by means of the PANSS and CGI scales. The scales were administered at hospital admission, after 3 weeks, 5 weeks, and at 4-weeks follow-up.

**Results:**

At the admission the patient PANSS total score was 136, the CGI score of 7. Aripiprazole was started and up-titrated to 30 mg/day. After 3 weeks, the positive symptoms were significantly reduced; due to the persistence of negative symptoms, clozapine 100 mg/day was added. At week 5 the PANSS total score decreased to 81. Due to poor insight we proposed aripiprazole LAI with the two-injection start. One month later, global functioning and illness insight improved; PANSS score was 43, CGI score 2. There was no evidence of akathisia or other side effects.

**Conclusions:**

Aripiprazole LAI showed good efficacy and tolerability in an adolescent with schizophrenia. The two-injection start regimen was a safe and viable option.

**Disclosure:**

No significant relationships.

